# Supplementary material for: Carbothermal Synthesis of Ni/Fe Bimetallic Nanoparticles Embedded into Graphitized Carbon for Efficient Removal of Chlorophenol
Source: Nanomaterials (Basel). 2021 May 27;11(6):1417. doi: 10.3390/nano11061417 (PMC8226776; doi:10.3390/nano11061417)
Supplement: Supplementary file 1 [file nanomaterials-11-01417-s001.zip › nanomaterials-1197388-supplementary.pdf]

nanomaterials

Supplementary Materials

Carbothermal Synthesis of Ni/Fe Bimetallic Nanoparticles Embedded into Graphitized Carbon for Efficient Removal of Chlorophenol

Min Zhuang [1,2], Wen Shi [2], Hui Wang [2,*], Liqiang Cui [2], Guixiang Quan [2] and Jinlong Yan [1,2,*]

1 School of the Environment and Safety Engineering, Jiangsu University, Zhenjiang 2120131, China; z17802592726@163.com
2 School of Environmental Science and Engineering, Yancheng Institute of Technology, Yancheng 224051, China; sw18351278375@126.com (W.S.); cuiliqiang411@163.com (L.C.); qgx@ycit.cn (G.Q.)
* Correspondence: whsl@ycit.cn (H.W.); yjlyt@yict.cn (J.Y.)

Electronic Supplementary Information

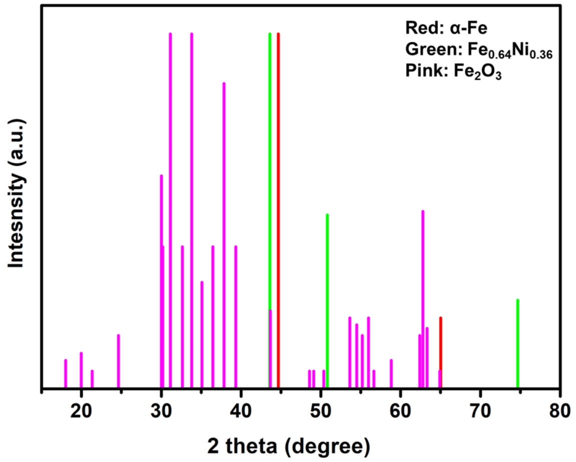

Figure S1. Standard X-ray diffraction patterns of α-Fe (PDF#06-0696), $Fe_{0.64}Ni_{0.36}$ (PDF#47-1405)and Fe_2O_3 (PDF#40-1139).

Copyright: © 2021 by the authors. Licensee MDPI, Basel, Switzerland. This article is an open access article distributed under the terms and conditions of the Creative Commons Attribution (CC BY) license (http://creativecommons.org/licenses/by/4.0/).

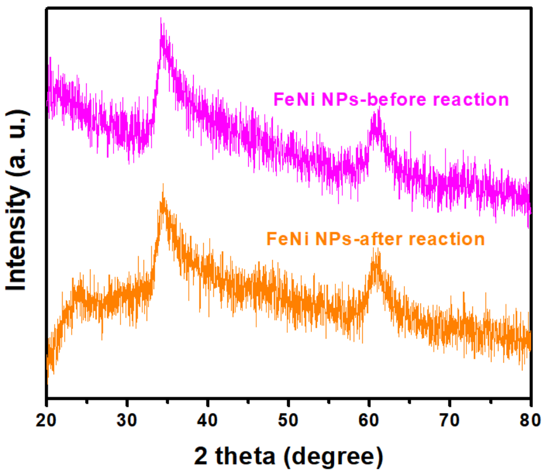

Figure S2. X-ray diffraction patterns of FeNi NPs before and after reaction.

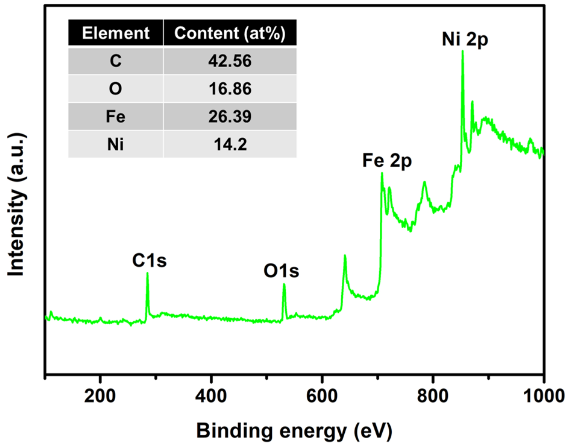

Figure S3. The X-ray photoelectron spectroscopy survey of NiFe@GC-50.

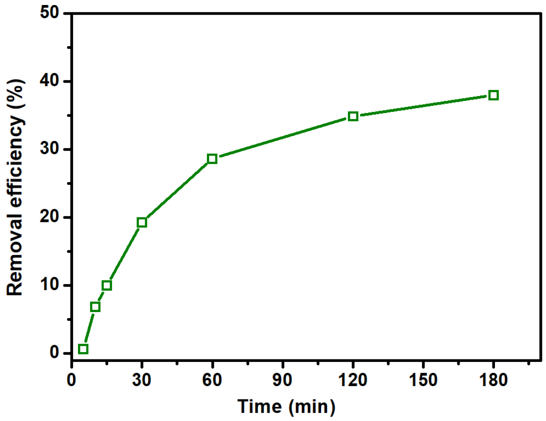

Figure S4. The profiles of 2,4,6-trichlorophenol (TCP) removal by graphitized carbon. (Experimental conditions: [TCP]=30 mg L^{-1}, pH=3.0, dosage=1.0 g L^{-1}, T=298.15 K).
